# Various aspects of piscine toxicology

**DOI:** 10.2478/v10102-010-0020-4

**Published:** 2010-09

**Authors:** Teresa Wlasow, Krystyna Demska-Zakes, Piotr Gomulka, Sylwia Jarmolowicz

**Affiliations:** 1 Faculty of Environmental Sciences and Fisheries, University of Warmia and Mazury in Olsztyn, Oczapowskiego 5, Olsztyn, Poland; 2 Inland Fisheries Institute, Oczapowskiego 10, Olsztyn, Poland

**Keywords:** ammonia, anesthetics, fish reproduction disruptors, oxidative disinfectants, phenol, toxicity

## Abstract

In opposition to toxicology of mammals piscine toxicology is closely connected with the conditions of external environment. The aquatic environment is necessary for embryonic development and after hatching during short or long-lasting larval period of most fish species. An aquatic environment is polluted by many industrial and agricultural wastes. Ammonia as a toxic and common compound in water have negative influence for aquaculture especially in intensive fish culture, recirculation system and hatchery facilities. Acute toxicity of ammonia was investigated in carp *Cyprinus carpio* L. and developmental stages of chub *Squalius cephalus* L. Changes in the peripheral blood characteristics and hemopoietic tissues of carp occurred after exposition to ammonia in acute tests and 3, 5 and 10 weeks sublethal concetration. The observed increase of the concentration of most amino acids in fish intoxicated with amonia suggests that the process reflects detoxication of ammonia which takes place both in the brain and muscles after 3 weeks of exposition. Phenol intoxication tests induced considerable unfavorable changes in the blood and dystrophic and necrobiotic lesions in tissues of fish leading to dysfunction both hemopoietic and reproductive processes.

In study on fish reproduction disruptors the influence of oxygenated polycyclic hydrocarbons (17-β-estradiol, 4,7-dihydroxyisoflavone, 1,6-dihydroxynaphthalene and 1,5-dihydroxynaphthalene) and oxygenated monocyclic hydrocarbons (phenol, 4-n-heptylphenol, 4-n-buthylphenol, 4-sec-buthylphenol; 4-tert-buthylphenol) was assessed using histopathological methods. It was established that examined oxygenated aromatic hydrocarbons both natural (17-β-estradiol and 4,7-dihydroxyisoflavone) and synthetic can disrupt the differentiation of primary and secondary sex traits in pikeperch *Sander lucioperca* L. The chronic activity of these “biomimetics of estrogen” can lead to the disappearance of natural fish population. *In vivo* and *in vitro* tests were used to exam dibutyl phthalate and butyl benzyl phthalate impact on the development of the reproductive system of pikeperch. Additional as multigenerational studies are needed to clarify influence long term exposure of fish to environmental concentrations of endocrine disrupting chemicals.

Hydrogen peroxide used in fish therapy is known to be toxic for sensitive species. In our work safe concentrations and exposure times was evaluated for ide *Leuciscus idus* L. and pike *Esox lucius* L. fry. The intensity of lesions in gills, skin, pseudobranch and thymus of exposed fish were connected with the time of bath.

Actually anesthetics are routinely required during stressful procedures with fish, but data regarding the safety of individual anesthetics to different fish species are still few and insufficient. The influence of clove oil, MS-222 and 2-phenoxyaethanol anesthesia on fish organism was investigated in our faculty with cooperation with Research Institute of Fish Culture and Hydrobiology, Vodnany, Czech Republic.

## Introduction

Toxicology is an interdisciplinary science, based on the knowledge and methods of miscellaneous fields. Fish toxicology, which refers to a piscine organism, covers the areas from a genome to a fish population. Scientific studies that consider fish as markers of environment contamination are placed within the environmental toxicology (Chan, 1995; Cho *et al*., 2009; Havelkova *et al*., 2007; Kovarova & Svobodova, 2009; Vethaak & Rheinallt, 1992). Among others, the knowledge about ichthyology and ichthyopathology is necessary here, as well as about analysis of biochemical indices and determination of xenobiotic residues in tissues. On the other hand, a level of these residues in edible fish tissues and their monitoring are important for human health protection (Brucka-Jastrzębska & Protasowicki, 2006; Protasowicki, 1987). In Poland, the content analyses of these components in muscles of fish intended for consumption are performed by the Toxicology Department of the National Veterinary Research Institute in Pulawy. The Institute also performs the research on kinetics of some poisons in fish organisms (Mitrowska *et al*., 2008).

Piscine toxicology can be considered as a part of veterinary toxicology and it deals with both farm and wild animals. In reports on intoxication cases among animals, fish poisonings are included besides poisonings of bees and farm animals, however, causes of fish intoxication are more difficult to explain as compared to terrestrial animals. About 50% of lethal cases among fishes remain unexplained due to many difficulties: delayed environmental and fish sampling, disappearance or translocation of a toxic substance in a contamination place, changes in tissues of dead fish that proceed faster than in terrestrial animals (Modra & Svobodova, 2009).

Mortal losses among fishes and other aquatic organisms observed in water reservoirs, even the sudden ones; do not always provide the information about contamination. They could be a consequence of serious diseases; therefore caution in the assessment is necessary. Numerous cases of mortality among native crayfish species in Poland were often associated with crayfish plague without any thorough research and without analysis of other causes (Kossakowski, 1973). Many fish disease entities caused by viruses, bacteria and parasites could constitute a cause of high mortality among species susceptible to infections. In this case, the aquatic environment and interactions are significant: toxins – pathogen, toxins – fish and its defense system, toxins – hydrobionts other than fish, pathogen – fish. The Polish ichthyopathologist, Professor Śnieszko, drew attention to complex determinants of a disease development in fish. Significance of the external environment and viruses, taking the stimulating and inhibiting factors into consideration, the development of pathological changes is presented by the hypothesis on the development of skin tumours in fish (Anders & Yoshimizu, 1994). The significance of environmental pollution for the development of diseases of aquatic organisms in the freshwater and marine environment (Sindermann, 1979, Svobodova *et al*., 1993) is still valid. Some effects of the influence exerted by xenobiotics on fish organisms in the development of neoplastic changes (Baumann, 1998,) or reproduction disorders (Popek *et al*., 2006; Simpson *et al*., 2000) evoke certain anxiety, not only about the future of fish, but also about the future of higher vertebrates, including humans (McAloose & Newton, 2009). New challenges in toxicology of fish are presented by nanotechnology and its products, which are within the interest of nanotoxicology (Laban, 2010). For some time now, the fish are also used as a model organism in biomedical studies (Belyaeva *et al*., 2009), which is yet another aspect of piscine toxicology.

This paper aims at presenting certain issues of piscine toxicology in order to illustrate different aspects of researches carried out at the Faculty of Environmental Sciences and Fisheries (FES&F), the University of Warmia and Mazury in Olsztyn. The present-day Faculty is derived from the Faculty of Fishery at the Agricultural College in Olsztyn, which was created in 1951. During the years 1966–1968, the departments specializing in marine subjects were moved to the University of Agriculture in Szczecin (at present, the West Pomeranian University of Technology), where the Faculty of Marine Fisheries and Food Technology were established. In the structure of FES&F in Olsztyn, we have 7 departments: Department of Applied Ecology, Department of Environmental Biotechnology, Department of Environmental Microbiology, Department of Environment Protection Engineering, Department of Fish Biology and Pisciculture, Department of Ichthyology and Department of Lake and River Fisheries without a separate unit for fish toxicology. The ongoing research projects in fish toxicology are related to current problems in fishery and aquaculture.

## The toxicity of phenol

As opposed to toxicology of mammals, fish toxicology is dominated by a strong relationship between the response of organisms and environmental conditions, as well as habitat preferences of specific fish species. Therefore, reactions of thermophilous species of cyprinids and coldwater salmonid fish may differ from each other. Higher sensitivity to phenol was observed among breams *Abramis brama* L. in winter (Waluga, 1975), whereas among specimens of rainbow trout *Oncorhynchus mykiss* Walbaum – during a warmer period (Wlasow, 1980). The choice of phenol as a toxicant in the experimental works carried out at the Department was due to the presence of this compound in the pollution of two main Polish rivers – the Vistula and the Oder. In subacute tests, applying the histopathological and hematological methods, the effect of phenol on blood and the hematopoietic system was assessed, as well as on the conditions of organs and tissues of bream – the most commonly occurring fish, (Waluga, 1966a; b). Analysis of long-term (3 months) influence by a low concentration of phenol (0.5 mg/dm^3^) was a subsequent stage. In these conditions, chronic intoxication without clear clinical symptoms was obtained (Waluga, 1975). Direct influence of phenol on a bream's organism can be described as necrogenic. Most acute necrobiotic changes affected the gills – a respiratory organ, which in fish is exposed to continuous and direct contact with water and elements contained in it. Less distinct necrobiotic changes affected the alimentary tract and the skin. In the indirect effect of low phenol concentration, properties of a system poison were revealed – dystrophic and necrobiotic changes in the hematopoietic and reproductive systems. The subacute intoxication proceeds without any local reaction with circulatory disturbances and with extensive necrotic foci. In prolonged intoxication of bream, nonspecific defense reactions were observed (Waluga, 1975). When investigating the influence of prolonged phenol intoxication on the rainbow trout (Wlasow, 1984, 1985a; b), changes in erythrocytic indices were determined, as well as the activity of selected enzymes and the dynamics of osmotic resistance of erythrocytes. Determination of cytological changes in the blood and in hemopoietic organs (splenograms and nephrograms) enabled to draw a conclusion about the process of organ metaplasia. For the first time in Poland, the lymphoblastic transformation test was applied for the fish, which reflects the ability of an intoxicated organism for cellular response, which is very common among fishes.

## Fish reproduction disruptors

Disorders in the reproductive system of fish, induced by substances occurring in the environment, raise interests among ichthyologists studying the causes of fish extinction in open waters. In the experiment on bream, fishes were exposed to phenol in the water (Waluga, 1975). In the experiment performed on pikeperch *Sander lucioperca*, 4-nonylphenol – a compound that accumulates in tissues of fish and other aquatic organisms, and analysed with different methods (Gadzała-Kopciuch *et al*., 2009), was dosed via a digestive track reaching a significant decrease in the contribution of males and the presence of bisexual fish (Demska-Zakes, 2004). The analysed compound did not cause any increase in mortality or in the growth index. In mature males, the quality of semen was reduced (Popek *et al*., 2006). With the exposure of pikeperch with undifferentiated gonads (60 days after hatching) to natural exoestrogens occurring in water – animal 17-β-estradiol and plant – 4,7-dihydroxyisoflavone, as well as synthetic compounds: oxygen derivatives of polycyclic hydrocarbons 1,6-dihydroxynaphthalene and 1,5-dihydroxynaphthalene and oxygen derivatives of monocyclic hydrocarbons – phenol, 4-n-heptyloxyphenol, 4-n-butylphenol, 4-sec-butylphenol, 4-tert-butylphenol – disturbances were being observed in the process of differentiation of the primary and secondary sex characteristics with the use of histological methods (Demska-Zakes, 2005). Oxygen derivatives of aromatic hydrocarbons can disturb the differentiation of primary and secondary sex characteristics of fish, whereas their chronic activity can bring about the change in the sex structure and extinction of fish. The extent of sterilization of testes or feminization depended on a compound and its concentration (the studied range 1–200 µg/dm^3^). Based on the lowest effective concentration, it was assessed that the highest activity is demonstrated by natural steroids – 17-β-estradiol and 4,7-dihydroxyisoflavone, whereas the lowest one - by xenobiotics with small molecular mass, *i.e.* phenol and butylphenol. Concentrations above 10 µg/dm^3^ reduce the survival rate, the growth rate and the condition of tested fishes (Demska-Zakes, 2005).

Phthalates – classified within the group of endocrine disrupters and produced in significant quantities – are known as substances that induce disorders in the function of endocrine glands of mammals. There is no information, however, about their influence on the fish reproductive system. The influence of dibutyl phthalate and butyl benzyl phthalate on the development of the reproductive system of pikeperch was analysed in the PhD thesis prepared at the Department of Ichthyology (Jarmolowicz, 2010).

## Ammonia

Ammonia is one of the most frequent lethal factors of fish intoxication (Modra & Svobodova, 2009), and can constitute problems and threat to fish in intensive breeding, the recirculation system and in hatcheries. In our research, the effect of ammonium compounds on a carp's organism was analysed, based on haematological and biochemical indices, as well as on cytological analysis of hemopoietic organs (Waluga & Flis, 1971; Dabrowska & Wlasow, 1986; Wlasow & Dabrowska, 1989; 1990; Wlasow *et al*., 1990). In acute tests (48 h LC_50_ for ammonium chloride 1.78 mg NH_3_/dm^3^ and ammonium nitrate 1.44 mg NH_3_/dm^3^) the lethal effect of both chemicals was similar but ammonium nitrate seems to be a stronger stressor. The sublethal concentration of ammonia (0.102 ± 0.059 mg NH_3_/dm^3^) induced unfavorable changes in the blood of carp. The observed increase of the concentration of most amino acids in fish intoxicated with amonia suggests that the process reflects detoxication of ammonia, which takes place both in the brain and muscles after 3 weeks of exposure. At present, researches are being carried out on the toxicity of ammonia in an early developmental stage of chub *Squalius cephalus* L.

## Hydrogen peroxide toxicity

After implementation of restrictions in the application of fish treatment agents (exclusion of malachite green), the necessity arose to investigate the compounds recognized as safe for fish and the environment. The research was carried out on the toxicity and the influence of hydrogen peroxide on an organism of early developmental stages of ide *Leuciscus idus* and pike *Esox lucius* L. (Wlasow *et al*., 2003a; b; 2004). Safe concentrations were determined, as well as the bath application time. Sensitivity of organs was demonstrated, including a thymus, which is exposed to environmental factors. When investigating the influence of hydrogen peroxide on a pike organism, the attention was paid to a pseudobranch – an organ, which is very seldom analyzed in toxicological studies. This organ plays an important role in the sight of fish, and thus its condition is extremely important for the fish that use eyesight when searching for food.

## Anesthetics

Anesthesia, euthanasia and sedation of both wild and captive fish are common requirements in aquaculture and fisheries research around the world. These clinical techniques facilitate a wide variety of activities such as sorting, grading, transportation, tagging, gamete collections, health monitoring, weight/length measurements, blood sampling and invasive surgery to name a few (Gomulka & Antychowicz, 1999).

Only several chemicals are used as fish anesthetics today, namely 2-phenoxyaethanol, etomidate, metomidate, eugenol and MS-222. The influence of eugenol and MS- 222 on blood indices of the Siberian sturgeon *Acipenser baeri* was investigated (Gomulka *et al*., 2008). We also tested effectiveness and safety of anesthetics commonly used in aquaculture for some other sturgeon species (Gomulka, 2008).

Modern aquaculture faces the need for diversification of aquaculture production to meet consumer's requirements. New fish species are introduced to intensive production. Following that, we have to better understand the influence of chemicals used in aquaculture on physiology of these species. The effect of clove oil and 2-phenoxyaethanol on blood indices of the European catfish *Silurus glanis* was studied too (Velišek *et al*., 2006; 2007).

In all countries with animal care legislation, anesthetics are routinely required during procedures that are deemed stressful or painful to fish. However, data regarding the safety of individual anesthetics for different fish species, especially in the case of endangered fish, such as loach *Misgurnus fossilis*, are still few and insufficient (Gomulka *et al*., 2007).
